# 1,3-Bis(pyridin-2-yl)-1*H*-benzimidazol-3-ium tetra­fluoridoborate

**DOI:** 10.1107/S1600536811027942

**Published:** 2011-07-16

**Authors:** Gabriele Grieco, Olivier Blacque, Heinz Berke

**Affiliations:** a Institute of Inorganic Chemistry, University of Zürich, Winterthurerstrasse 190, 8057 Zürich, Switzerland

## Abstract

The asymmetric unit of the title compound, C_17_H_13_N_4_^+^·BF_4_^−^, contains one half of the benzimidazolium cation and one half of the tetra­fluoridoborate anion, with crystallographic mirror planes bis­ecting the mol­ecules. One F atom of the tetra­fluoridoborate is equally disordered about a crystallographic mirror plane. In the crystal, C—H⋯F inter­actions link the cations and anions into layers parallel to (100). The crystal packing is further stabilized by F⋯π contacts involving the tetra­fluoridoborate anions and the five-membered rings [F⋯centroid = 2.811 (2) Å].

## Related literature

For applications of *N*,*N′*-bis­(2-pyrid­yl)aryl­diamines, see: Stoessel *et al.* (2010 ▶); Goldfarb (2009 ▶) and of imidazolium salts, see: Berlin *et al.* (2007 ▶); Bold *et al.* (2005 ▶); Huang *et al.* (2005 ▶); Murakami *et al.* (2007 ▶); Teles *et al.* (1996 ▶). For pharmaceuticals based on the aniline–pyridine scaffold, see: Kim *et al.* (1996 ▶); Wu *et al.* (2001 ▶). For the synthesis of the starting material *N*,*N*′-bis­(pyridin-2-yl)benzene-1,2-diamine, see: Gdaniec *et al.* (2004 ▶).
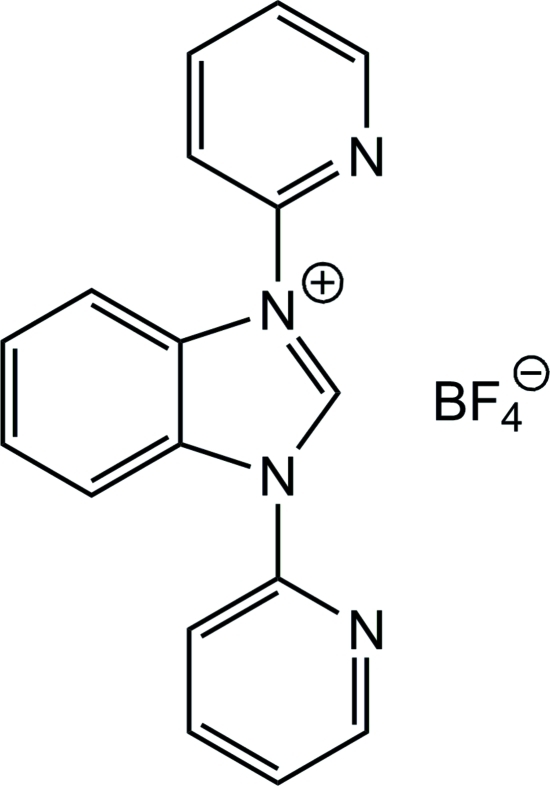


## Experimental

### 

#### Crystal data

C_17_H_13_N_4_^+^·BF_4_^−^
*M* *_r_* = 360.12
Orthorhombic, 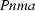

*a* = 7.3412 (2) Å
*b* = 17.5051 (5) Å
*c* = 12.2426 (3) Å
*V* = 1573.28 (7) Å^3^
*Z* = 4
Mo *K*α radiation
μ = 0.13 mm^−1^
*T* = 183 K
0.44 × 0.31 × 0.11 mm

#### Data collection

Oxford Diffraction Xcalibur diffractometer with a Ruby detector
Absorption correction: analytical [*CrysAlis PRO* (Oxford Diffraction, 2010 ▶) based on Clark & Reid (1995 ▶)] *T*_min_ = 0.964, *T*_max_ = 0.991
8816 measured reflections
2014 independent reflections
1543 reflections with *I* > 2σ(*I*)
*R*_int_ = 0.022

#### Refinement

*R*[*F* ^2^ > 2σ(*F* ^2^)] = 0.042
*wR*(*F* ^2^) = 0.117
*S* = 1.08
2014 reflections
130 parameters
H atoms treated by a mixture of independent and constrained refinement
Δρ_max_ = 0.27 e Å^−3^
Δρ_min_ = −0.36 e Å^−3^

### 

Data collection: *CrysAlis PRO* (Oxford Diffraction, 2010 ▶); cell refinement: *CrysAlis PRO*; data reduction: *CrysAlis PRO*; program(s) used to solve structure: *SHELXS97* (Sheldrick, 2008 ▶); program(s) used to refine structure: *SHELXL97* (Sheldrick, 2008 ▶); molecular graphics: *Mercury* (Macrae *et al.*, 2006 ▶), *ORTEP-3 for Windows* (Farrugia, 1997) ▶ and *POV-RAY for Windows* (Cason, 2003 ▶); software used to prepare material for publication: *WinGX* (Farrugia, 1999 ▶) and *publCIF* (Westrip, 2010 ▶).

## Supplementary Material

Crystal structure: contains datablock(s) global, I. DOI: 10.1107/S1600536811027942/su2291sup1.cif

Structure factors: contains datablock(s) I. DOI: 10.1107/S1600536811027942/su2291Isup2.hkl

Supplementary material file. DOI: 10.1107/S1600536811027942/su2291Isup3.cml

Additional supplementary materials:  crystallographic information; 3D view; checkCIF report

## Figures and Tables

**Table 1:** Hydrogen-bond geometry (Å, °)

*D*—H⋯*A*	*D*—H	H⋯*A*	*D*⋯*A*	*D*—H⋯*A*
C1—H1⋯F3	0.95 (2)	2.14 (2)	3.094 (2)	178 (2)
C9—H9⋯F1^i^	0.93	2.62	3.4759 (19)	154

Symmetry code: (i) 
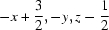
.

**Fig. 1.**
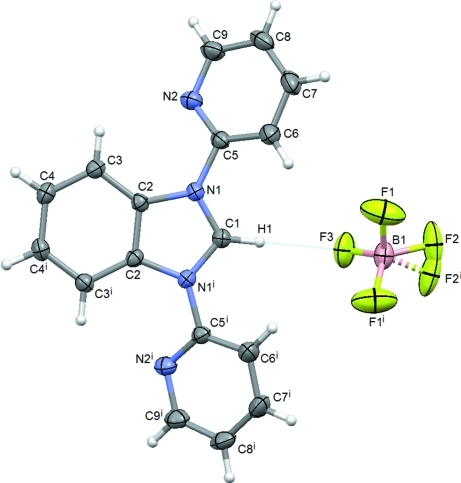
The molecular structure of the title compound, with the numbering scheme and displacement ellipsoids drawn at the 50% probability level (H-atoms are shown as spheres of arbitrary size). Symmetry code: (i) -x + 3/2, -y, z - 1/2.

**Fig. 2.**
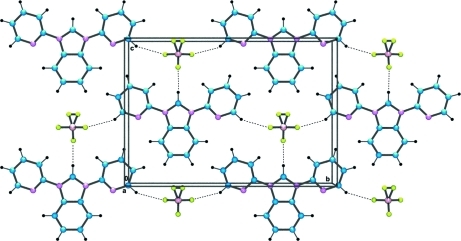
View normal to (100) of the two-dimensional network of the title compound, formed by intermolecular C—H···F interactions (dashed lines; see Table 1 for details).

**Fig. 3.**
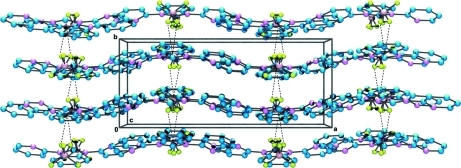
View normal to (001) of the three-dimensional network of the title compound, formed by intermolecular B—F···π interactions (dashed lines; see Comment section for details).

**Fig. 4.**
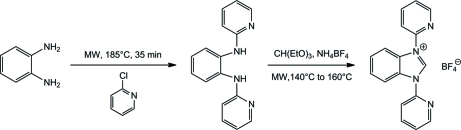
Reaction scheme.

## supplementary crystallographic information

### Comment

*N*,*N'*-bis(2-pyridyl)aryldiamines are an important class of compounds that are useful as intermediates in the syntheses of organic electroluminescent devices (LED) (Stoessel *et al.*, 2010), or as compounds useful for altering the lifespan of eukaryotic organisms in the yeast (Goldfarb, 2009). Imidazolium salts based on the benzoimidazole scaffold are used in electronics (Bold *et al.*, 2005; Murakami *et al.*, 2007) and in catalytic processes involving the use of the benzimidazolium salt as it is (Teles *et al.*, 1996), or bound to a metal (Berlin *et al.*, 2007; Huang *et al.*, 2005).

We used the *N*,*N'*-bis(2-pyridyl)-benzene-1,2-diamine compound as a starting material for the synthesis of the title compound, a new imidazolium salt. Despite the fact that the synthesis of the starting material *N*,*N'*-bis(pyridin-2-yl)benzene-1,2-diamine had been reported previously (Gdaniec *et al.*, 2004), we optimized the procedure to obtain a better yield (91 instead of 70%) using the reaction reported in the Experimental section (Fig. 4) with a microwave technique. A similar procedure was succesfully used to synthesize the title compound in a very high yield (99.7%). It is the first example of a coupling between a halo pyridine and an aniline made with microwave heating that does not imply the use of any metal. This method to couple halo pyridines and anilines can be very useful to produce compounds with pharmaceutical activity since many pharmaceuticals are based on the aniline-pyridine scaffold (Kim *et al.*, 1996; Wu *et al.*, 2001).

The asymmetric unit of the title compound, C_17_H_13_N_4_^+^.BF_4_^-^, contains one half-molecule of the benzimidazolium cation and one half-molecule of the anion, crystallographic mirror planes bisecting the molecules (Fig. 1). One F atom of the tetrafluoroborate is disordered over two positions. The second position being generated by a crystallographic mirror plane, the site-occupancy factor is 0.5. The benzimidazole and pyridine rings are not coplanar, the dihedral angle between the mean planes is 26.67 (4)°.

In the crystal intermolecular C—H···F interactions link the cations and anions into layers parallel to the (100) crystallographic plane (Fig. 2, Table 1). The crystal packing is further stabilizes by F···π contacts (Fig. 3) involving the tetrafluoridoborate anions and the five-membered rings of the benzimidazole rings [F···centroid = 2.811 (2) Å].

### Experimental

To benzene-1,2-diamine (2.7 g, 24.97 mmol) in a microwave vial, 2-chloropyridine (9 ml, 46.95 mmol) was added. The vial was then closed with a cap consisting of a Teflon septum and the reaction mixture was heated for 35 mins at 458 K. Monitoring with TLC (thin layer chromatography) and GC—MS (gas chromatography - mass spectrometry) showed that the benzene-1,2-diamine was consumed after this time and the mixture was allowed to cool to room temperature. The crude mixture was dissolved in water (15 ml) and dropped into a solution of concentrated ammonia in water (25 ml of NH_4_OH 24.5% in 250 ml of water). The resulting pink-red precipitate was filtered off, washed with water (50 ml) and was subsequently dried in air to give *N*,*N'*-bis(pyridin-2-yl)benzene-1,2-diamine. Further recrystallization from ethanol gave a very pure product (yield: 2.902 g, 91%).

To *N*,*N'*-bis(pyridin-2-yl)benzene-1,2-diamine (500 mg, 1.91 mmol) in a microwave vial, finely ground ammonium tetrafluoroborate (204 mg, 1.91 mmol) was added followed by the triethyl orthoformate (10 ml, 59 mmol). The vial with the red pink suspension was then closed with a cap consisting of a Teflon septum and the reaction mixture was heated for 25 minutes at 413 K and then for further 20 minutes to 433 K. After that time inside the vial a blue-violet solid was present and TLC analysis showed that the starting material had been consumed. The solid was separated off and then stirred with ethyl acetate (3x50 ml) for 15 minutes. It was then collected by suction filtration, washed with diethyl ether and dried to afford a deep violet-blue compound (yield: 686 mg, 99.7%). Recrystallization from a hot saturated solution of water/methanol (9/2) afforded red plate-like crystals of the title compound, suitable for X-ray analysis. Spectroscopic data for the title compound are given in the archived CIF.

### Refinement

One F atom of the tetrafluoroborate ion is disordered over two positions around a mirror plane (site-occupancy factor of 1/2). H-atom H1 was located in a difference Fourier map and was freely refined. The C-bound H-atoms were included in calculated positions and treated as riding atoms: C—H = 0.93 Å with *U*_iso_(H) = 1.2*U*_eq_(C).

### Crystal data

**Table:**

C_17_H_13_N_4_^+^·BF_4_^−^	*F*(000) = 736
*M**_r_* = 360.12	*D*_x_ = 1.52 Mg m^−^^3^
Orthorhombic, *P**n**m**a*	Mo *K*α radiation, λ = 0.71073 Å
Hall symbol: -P 2ac 2n	Cell parameters from 4054 reflections
*a* = 7.3412 (2) Å	θ = 2.8–32.6°
*b* = 17.5051 (5) Å	µ = 0.13 mm^−^^1^
*c* = 12.2426 (3) Å	*T* = 183 K
*V* = 1573.28 (7) Å^3^	Plate, red
*Z* = 4	0.44 × 0.31 × 0.11 mm

### Data collection

**Table:**

Oxford Diffraction Xcalibur diffractometer with a Ruby detector	1543 reflections with *I* > 2σ(*I*)
ω scans	*R*_int_ = 0.022
Absorption correction: analytical [*CrysAlis PRO* (Oxford Diffraction, 2010) based on expressions derived by Clark & Reid (1995)]	θ_max_ = 28.3°, θ_min_ = 3.2°
*T*_min_ = 0.964, *T*_max_ = 0.991	*h* = −9→9
8816 measured reflections	*k* = −20→23
2014 independent reflections	*l* = −12→16

### Refinement

**Table:**

Refinement on *F*^2^	0 restraints
Least-squares matrix: full	H atoms treated by a mixture of independent and constrained refinement
*R*[*F*^2^ > 2σ(*F*^2^)] = 0.042	*w* = 1/[σ^2^(*F*_o_^2^) + (0.062*P*)^2^ + 0.2418*P*] where *P* = (*F*_o_^2^ + 2*F*_c_^2^)/3
*wR*(*F*^2^) = 0.117	(Δ/σ)_max_ < 0.001
*S* = 1.08	Δρ_max_ = 0.27 e Å^−^^3^
2014 reflections	Δρ_min_ = −0.36 e Å^−^^3^
130 parameters	

### Special details

**Table:**

Experimental. Spectroscopic data for the title compound:^1^H-NMR in CD_3_CN: δ = 10.07 (s, 1H), δ = 8.837 (dd, 2H), δ = 8.48 (m, 2H), δ = 8.29 (m,2H), δ = 8.04 (t, 1H), δ = 8.01 (t, 1H), δ = 7.87 (m, 2H) δ = 7.77 (m, 2H).^13^C-NMR in CD_3_CN: δ = 150.95, 141.60, 129.49, 126.93, 118.83, 118.26,116.86.^19^F-NMR in CD_3_CN: δ = -152.32.
Geometry. All e.s.d.'s (except the e.s.d. in the dihedral angle between two l.s. planes) are estimated using the full covariance matrix. The cell e.s.d.'s are taken into account individually in the estimation of e.s.d.'s in distances, angles and torsion angles; correlations between e.s.d.'s in cell parameters are only used when they are defined by crystal symmetry. An approximate (isotropic) treatment of cell e.s.d.'s is used for estimating e.s.d.'s involving l.s. planes.
Refinement. Refinement of *F*^2^ against ALL reflections. The weighted *R*-factor *wR* and goodness of fit *S* are based on *F*^2^, conventional *R*-factors *R* are based on *F*, with *F* set to zero for negative *F*^2^. The threshold expression of *F*^2^ > σ(*F*^2^) is used only for calculating *R*-factors(gt) *etc*. and is not relevant to the choice of reflections for refinement. *R*-factors based on *F*^2^ are statistically about twice as large as those based on *F*, and *R*- factors based on ALL data will be even larger.

### Fractional atomic coordinates and isotropic or equivalent isotropic displacement parameters (Å^2^)

**Table:**

	*x*	*y*	*z*	*U*_iso_*/*U*_eq_	Occ. (<1)
C1	0.7110 (2)	0.25	0.56060 (15)	0.0232 (4)	
C2	0.64692 (16)	0.21032 (7)	0.39260 (10)	0.0211 (3)	
C3	0.60860 (17)	0.16850 (8)	0.29898 (10)	0.0243 (3)	
H3	0.6088	0.1154	0.299	0.029*	
C4	0.57020 (17)	0.20992 (8)	0.20603 (11)	0.0263 (3)	
H4	0.5436	0.1841	0.1416	0.032*	
C5	0.69854 (17)	0.11073 (8)	0.53968 (11)	0.0245 (3)	
C6	0.6441 (2)	0.09450 (9)	0.64542 (12)	0.0336 (3)	
H6	0.6026	0.1324	0.6925	0.04*	
C7	0.6546 (2)	0.01864 (9)	0.67741 (13)	0.0397 (4)	
H7	0.622	0.0045	0.748	0.048*	
C8	0.7136 (2)	−0.03573 (9)	0.60414 (14)	0.0374 (4)	
H8	0.7192	−0.087	0.624	0.045*	
C9	0.7640 (2)	−0.01265 (8)	0.50081 (14)	0.0345 (4)	
H9	0.8038	−0.0496	0.4517	0.041*	
N1	0.68805 (14)	0.18768 (6)	0.49998 (9)	0.0223 (3)	
N2	0.75864 (17)	0.06028 (7)	0.46749 (10)	0.0302 (3)	
F1	0.58623 (16)	0.18658 (7)	0.89742 (12)	0.0763 (4)	
F2	0.8041 (3)	0.2711 (2)	0.98569 (15)	0.0680 (16)	0.5
F3	0.8073 (2)	0.25	0.80661 (11)	0.0577 (4)	
B1	0.6969 (3)	0.25	0.89769 (19)	0.0338 (5)	
H1	0.743 (3)	0.25	0.6360 (19)	0.024 (5)*	

### Atomic displacement parameters (Å^2^)

**Table:**

	*U*^11^	*U*^22^	*U*^33^	*U*^12^	*U*^13^	*U*^23^
C1	0.0249 (9)	0.0230 (10)	0.0217 (9)	0	−0.0010 (7)	0
C2	0.0210 (6)	0.0224 (7)	0.0200 (6)	0.0008 (5)	0.0019 (5)	0.0021 (5)
C3	0.0254 (6)	0.0229 (7)	0.0245 (7)	−0.0016 (5)	0.0012 (5)	−0.0029 (5)
C4	0.0263 (6)	0.0313 (7)	0.0212 (6)	−0.0013 (5)	0.0002 (5)	−0.0043 (5)
C5	0.0257 (6)	0.0215 (7)	0.0263 (7)	−0.0014 (5)	−0.0046 (5)	0.0030 (5)
C6	0.0450 (8)	0.0289 (8)	0.0269 (7)	−0.0052 (6)	−0.0002 (6)	0.0010 (6)
C7	0.0521 (9)	0.0371 (9)	0.0297 (7)	−0.0092 (7)	−0.0045 (7)	0.0114 (7)
C8	0.0416 (9)	0.0249 (8)	0.0457 (9)	−0.0009 (6)	−0.0092 (7)	0.0117 (7)
C9	0.0388 (8)	0.0235 (8)	0.0411 (8)	0.0055 (6)	−0.0036 (6)	0.0020 (6)
N1	0.0258 (5)	0.0211 (6)	0.0200 (5)	0.0001 (4)	−0.0006 (4)	0.0015 (4)
N2	0.0353 (6)	0.0230 (6)	0.0325 (6)	0.0033 (5)	0.0002 (5)	0.0019 (5)
F1	0.0558 (7)	0.0436 (7)	0.1295 (12)	−0.0010 (5)	0.0195 (7)	0.0143 (7)
F2	0.0400 (9)	0.132 (5)	0.0315 (9)	−0.0042 (12)	−0.0083 (7)	−0.0174 (14)
F3	0.0646 (10)	0.0810 (12)	0.0276 (7)	0	0.0114 (6)	0
B1	0.0284 (11)	0.0494 (16)	0.0236 (11)	0	−0.0016 (9)	0

### Geometric parameters (Å, °)

**Table:**

C1—N1	1.3302 (15)	C6—C7	1.387 (2)
C1—H1	0.95 (2)	C6—H6	0.93
C2—C3	1.3887 (18)	C7—C8	1.378 (2)
C2—C2^i^	1.389 (3)	C7—H7	0.93
C2—N1	1.4060 (16)	C8—C9	1.379 (2)
C3—C4	1.3784 (18)	C8—H8	0.93
C3—H3	0.93	C9—N2	1.3408 (19)
C4—C4^i^	1.403 (3)	C9—H9	0.93
C4—H4	0.93	F1—B1	1.3756 (18)
C5—N2	1.3249 (18)	F2—B1	1.384 (3)
C5—C6	1.384 (2)	F3—B1	1.379 (3)
C5—N1	1.4341 (17)		
			
N1—C1—N1^i^	110.20 (16)	C8—C7—H7	120.2
N1—C1—H1	124.88 (9)	C6—C7—H7	120.2
N1^i^—C1—H1	124.88 (9)	C7—C8—C9	118.64 (14)
C3—C2—C2^i^	121.81 (8)	C7—C8—H8	120.7
C3—C2—N1	131.81 (12)	C9—C8—H8	120.7
C2^i^—C2—N1	106.37 (7)	N2—C9—C8	123.39 (15)
C4—C3—C2	116.46 (13)	N2—C9—H9	118.3
C4—C3—H3	121.8	C8—C9—H9	118.3
C2—C3—H3	121.8	C1—N1—C2	108.53 (11)
C3—C4—C4^i^	121.74 (8)	C1—N1—C5	125.06 (12)
C3—C4—H4	119.1	C2—N1—C5	126.39 (11)
C4^i^—C4—H4	119.1	C5—N2—C9	116.19 (13)
N2—C5—C6	125.68 (13)	F1—B1—F1^i^	107.61 (19)
N2—C5—N1	114.70 (12)	F1—B1—F3	110.21 (13)
C6—C5—N1	119.61 (12)	F1—B1—F2^i^	97.01 (18)
C5—C6—C7	116.38 (14)	F1—B1—F2	123.6 (2)
C5—C6—H6	121.8	F1^i^—B1—F2	97.01 (18)
C7—C6—H6	121.8	F3—B1—F2	107.17 (18)
C8—C7—C6	119.69 (14)		

Symmetry codes: (i) *x*, −*y*+1/2, *z*.

### Hydrogen-bond geometry (Å, °)

**Table:**

*D*—H···*A*	*D*—H	H···*A*	*D*···*A*	*D*—H···*A*
C1—H1···F3	0.95 (2)	2.14 (2)	3.094 (2)	178.(2)
C9—H9···F1^ii^	0.93	2.62	3.4759 (19)	154

Symmetry codes: (ii) −*x*+3/2, −*y*, *z*−1/2.

## References

[bb1] Berlin, J. M., Campbell, K., Ritter, T., Funk, T. W., Chlenov, A. & Grubbs, R. H. (2007). *Org. Lett.* **9**, 1339–1342. 10.1021/ol070194o 17343392

[bb2] Bold, M., Lennartz, C., Prinz, M., Schmidt, H.-W., Thelakkat, M., Baete, M., Neuber, C., Kowalsky, W., Schildknecht, C. & Johannes, H.-H. (2005). PCT Int. Appl. WO 2005019373.

[bb3] Cason, C. J. (2003). *POV-RAY* Persistence of Vision Raytracer Pty. Ltd, Victoria, Australia.

[bb4] Clark, R. C. & Reid, J. S. (1995). *Acta Cryst.* A**51**, 887–897.

[bb18] Farrugia, L. J. (1997). *J. Appl. Cryst.* **30**, 565.

[bb5] Farrugia, L. J. (1999). *J. Appl. Cryst.* **32**, 837–838.

[bb6] Gdaniec, M., Bensemann, I. & Połoński, T. (2004). *Acta Cryst.* C**60**, o215–o216. 10.1107/S010827010400068X 15004387

[bb7] Goldfarb, D. S. (2009). US Patent Appl. Publ. US 20090163545.

[bb8] Huang, W., Guo, J., Xiao, Y., Zhu, M., Zou, G. & Tang, J. (2005). *Tetrahedron*, **61**, 9783–9790.

[bb9] Kim, H.-J., Han, Y.-H., Chung, S.-J., Lee, M.-H. & Shim, C.-K. (1996). *Arch. Pharm. Res.* **19**, 297–301.

[bb10] Macrae, C. F., Edgington, P. R., McCabe, P., Pidcock, E., Shields, G. P., Taylor, R., Towler, M. & van de Streek, J. (2006). *J. Appl. Cryst.* **39**, 453–457.

[bb11] Murakami, T., Yagi, K., Ichijima, S., Igarashi, T. & Satou, T. (2007). WO 2007034985.

[bb12] Oxford Diffraction (2010). *CrysAlis PRO* Oxford Diffraction Ltd, Yarnton, England.

[bb13] Sheldrick, G. M. (2008). *Acta Cryst.* A**64**, 112–122. 10.1107/S0108767307043930 18156677

[bb14] Stoessel, P., Heil, H., Joosten, D., Pflumm, C. & Gerhard, A. (2010). PCT Int. Appl. WO 2010099852.

[bb15] Teles, J. H., Melder, J.-P., Ebel, K., Schneider, R., Gehrer, E., Harder, W., Brode, S., Enders, D., Breuer, K. & Raabe, G. (1996). *Helv. Chim. Acta*, **79**, 61–83.

[bb16] Westrip, S. P. (2010). *J. Appl. Cryst.* **43**, 920–925.

[bb17] Wu, S. N., Jan, C. R. & Chiang, H. T. (2001). *J. Investig. Med.* **49**, 522–533. 10.2310/6650.2001.33629 11730088

